# Principal Networks

**DOI:** 10.1371/journal.pone.0060997

**Published:** 2013-04-22

**Authors:** Jonathan D. Clayden, Michael Dayan, Chris A. Clark

**Affiliations:** 1 Institute of Child Health, University College London, London, United Kingdom; 2 Neuroimaging Laboratory, Fondazione Santa Lucia, Rome, Italy; Cuban Neuroscience Center, Cuba

## Abstract

Graph representations of brain connectivity have attracted a lot of recent interest, but existing methods for dividing such graphs into connected subnetworks have a number of limitations in the context of neuroimaging. This is an important problem because most cognitive functions would be expected to involve some but not all brain regions. In this paper we outline a simple approach for decomposing graphs, which may be based on any measure of interregional association, into coherent “principal networks”. The technique is based on an eigendecomposition of the association matrix, and is closely related to principal components analysis. We demonstrate the technique using cortical thickness and diffusion tractography data, showing that the subnetworks which emerge are stable, meaningful and reproducible. Graph-theoretic measures of network cost and efficiency may be calculated separately for each principal network. Unlike some other approaches, all available connectivity information is taken into account, and vertices may appear in none or several of the subnetworks. Subject-by-subject “scores” for each principal network may also be obtained, under certain circumstances, and related to demographic or cognitive variables of interest.

## Introduction

There has been a substantial amount of recent interest in network representations of neural connectivity. Abstract “graphs” can be employed to represent the broad pattern of association between brain regions, using structural and functional imaging methods to obtain this information [1]; and a great deal of theory exists regarding the characterisation and manipulation of graphs in general, dating back as far as Leonhard Euler's 18th-century solution to the now-famous “seven bridges of Königsberg” problem [2]. This body of theory offers tools for describing and comparing patterns of brain interconnectivity, with potential relevance for clinical and nonclinical neuroscience. For example, one might expect that a network in which links between regions are laid out less “efficiently” would correspond to a penalty in mental processing speed—and early clinical and cognitive studies using graph theory are beginning to bear such views out (e.g. [3], [4]). Such characteristics are also useful in understanding the trade-offs associated with brain evolution under practical space and resource constraints [5]; and graphs are widely used in genomics and proteomics.

The abstract nature of graphs means that the same theoretical platform may be used to analyse networks generated from very different source data, and this may be seen as both an advantage and a potential source of confusion. Brain connectivity graphs have been created using measures of association such as between-subject correlations in cortical thickness [3], [6], within-subject correlations in functional imaging time series [7], [8], or the number of reconstructed streamlines from diffusion tractography [9], [10]. Attempts to unify the findings in these different modalities have tended to observe some similarities between the graphs, but also notable differences [11], [12]. The choice of brain regions to act as the vertices of the graphs tends to be ultimately arbitrary, but may be based on a semiautomated anatomical parcellation, or else simply by creating many small cortical patches of similar size. However, the choice of vertices, as well as the exact basis for retaining or discarding the edges which connect them, will have a significant impact on the results and conclusions of a given analysis [13].

In clinical and neuroscientific applications, the difficulties involved in reliably applying graph methods are compounded. The functional importance of the “small-world” organisation of the brain, with some regions forming highly connected hubs, is well recognised [9], [14], [15], and in any given study it is reasonable to assume that only a subset of the cortical regions obtained from a full brain parcellation will be of relevance—but graph-theoretical analysis is usually performed on the whole network. The best way to identify important subnetworks is not obvious: this is a problem which has received considerable attention over several decades in the broader network analysis literature, under the general heading of “community detection” [16]. This term generally refers to the process of analysing a graph and partitioning it into “communities” of highly interconnected vertices, often choosing the partition which maximises “modularity”, a measure which quantifies the excess within-community connectivity relative to a randomly connected graph. Modularity maximisation methods have been applied in a number of imaging studies (e.g. [17], [18]), but such methods tend to consider only whether each pair of nodes is connected, without taking the weight of each connection into account. As an alternative to modularity optimisation, some studies have performed hierarchical clustering to group strongly associated vertices into subgraphs (e.g. [19], [20]), a natural approach which does take advantage of the availability of a similarity measure between regions. But this technique does not directly indicate the relative importance of each subnetwork, and nor is it clear when one should stop agglomerating or dividing clusters. Finally, Iturria-Medina et al. [10] identify common “motifs” in their networks, but these are limited to patterns of interconnection between very small and nonspecific groups of vertices.

In this paper we introduce the concept of “principal networks”, a simple technique for identifying influential subnetworks with strong internal connectivity using a data-driven eigendecomposition approach, akin to principal components analysis (PCA). We present this framework as an alternative to graph partitioning methods, which groups together vertices with similar patterns of association and allows multiple “layers” of connectivity to be considered separately. The relative importance of different subnetworks can be easily established using this method, as can the degree of influence of each cortical region in each principal network. We demonstrate the method using cortical thickness and diffusion tractography data.

## Results

For brevity in the presentation of these results, each cortical region segmented by Freesurfer is given a numerical index. The correspondence between region names and indices is given in Table 1.

**Table 1:** Correspondence between region names and indices used in this paper.

Region name	Index (left)	Index (right)
caudal anterior cingulate cortex	1	33
caudal middle frontal gyrus	2	34
cuneus	3	35
entorhinal cortex	4	36
fusiform gyrus	5	37
inferior parietal gyrus	6	38
inferior temporal gyrus	7	39
cingulate gyrus, isthmus	8	40
lateral occipital cortex	9	41
lateral orbitofrontal cortex	10	42
lingual gyrus	11	43
medial orbitofrontal gyrus	12	44
middle temporal gyrus	13	45
parahippocampal gyrus	14	46
paracentral gyrus	15	47
inferior frontal gyrus, pars opercularis	16	48
inferior frontal gyrus, pars orbitalis	17	49
inferior frontal gyrus, pars triangularis	18	50
pericalcarine cortex	19	51
postcentral gyrus	20	52
posterior cingulate gyrus	21	53
precentral gyrus	22	54
precuneus	23	55
rostral anterior cingulate cortex	24	56
rostral middle frontal gyrus	25	57
superior frontal gyrus	26	58
superior parietal gyrus	27	59
superior temporal gyrus	28	60
supramarginal gyrus	29	61
frontal pole	30	62
temporal pole	31	63
transverse temporal gyrus	32	64

Note that each region has one index value for each of the left and right hemispheres.

**Figure 1:**
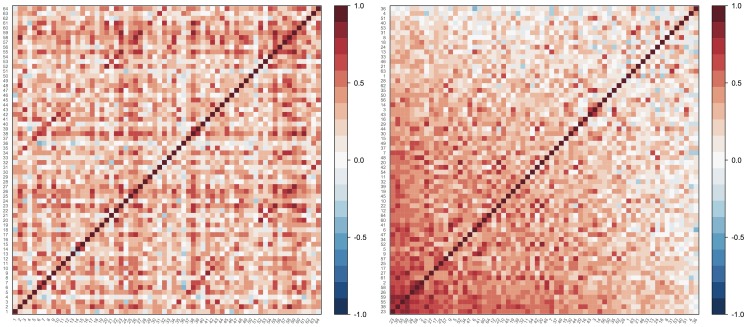
Visualisation of the full association matrix derived from all cortical thickness data. The matrix is shown twice, with the gyral regions ordered either numerically by index (left), or by their loading in the first principal network (right). The scale is based on Pearson's correlation coefficient between regions, across all participants.

The full association matrix derived from cortical thickness data from all 28 participants is shown in Fig. 1. We observe that it is dominated by moderately strong positive correlations but, by ordering the rows and columns according to their loading in the first principal network, the matrix appears more structured.

**Figure 2:**
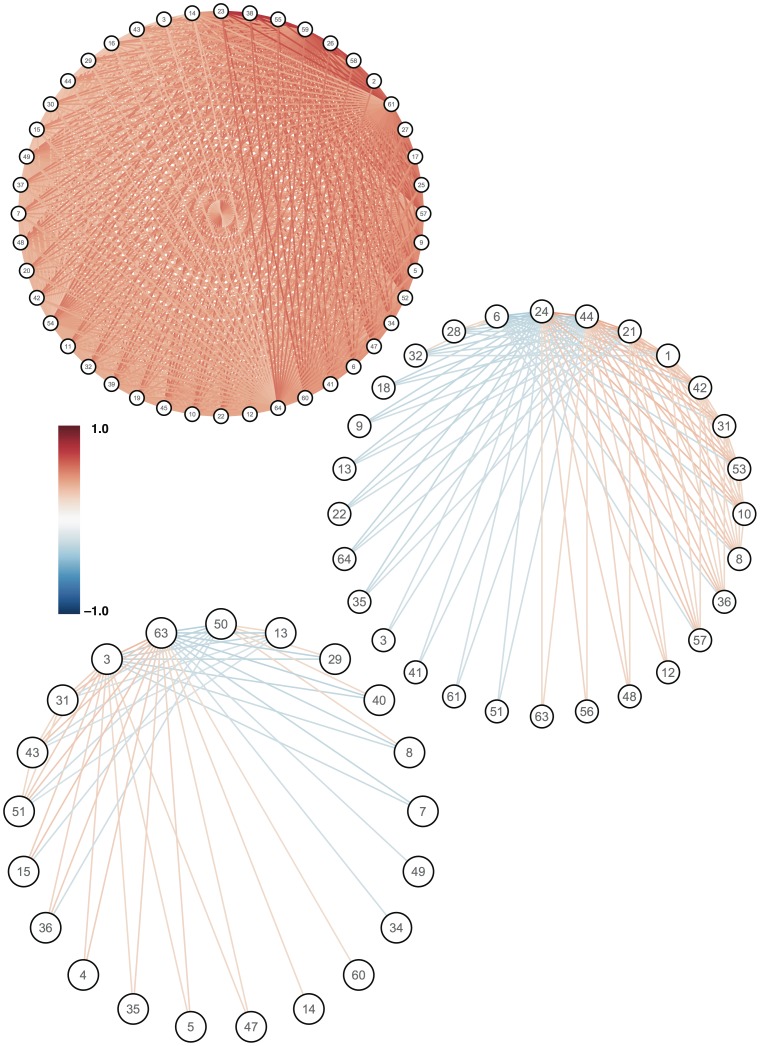
First (top), second (middle) and third (bottom) principal networks, based on all cortical thickness data. Only vertices and edges above the appropriate loading and weight thresholds are shown. The vertices are laid out regularly in a circle for visual clarity, and ordered by their loading in the appropriate PN.

Graphs representing the most influential three principal networks (PNs) are shown in Fig. 2. Note that since the association matrix in this case is a correlation matrix, the principal networks are equivalent to principal components of the original cortical thickness data. It is immediately evident that the first PN captures a broad tendency for positive correlation in cortical thickness between the majority of the regions of interest. It includes 44 of the 64 regions, and is fully connected; i.e. every vertex is connected to every other. By contrast, the second PN includes only 28 vertices and is far more sparsely connected. It also includes a mixture of positively and negatively weighted edges. Various graph-theoretical characteristics of the original graph, as well as all PNs containing at least two vertices, are given in Table 2. We note that the most strongly-connected vertex in both the full graph and PN1 is the left precuneus, an area regularly reported as being important in brain networks (see, for example, [9]).

**Table 2:** Graph characteristics of cortical thickness networks.

Principal network	Full network	1	2	3	4	5	6	7
**Eigenvalue**	N/A	21.55	6.47	5.16	4.07	3.18	2.52	2.40
**Number of connected vertices**	64	44	28	21	13	7	4	3
**Most connected vertex**	23	23	24	63	54	4	14	53
**Number of edges**	1434	990	113	56	36	7	10	5
**Connection density, %**	68.94	100.00	27.83	24.24	39.56	25.00	100.00	83.33
**Mean absolute edge weight**	0.44	0.43	0.25	0.24	0.26	0.23	0.23	0.22
**Mean shortest path, steps**	1.32	1.00	1.72	1.76	1.63	1.71	1.00	1.00
**Mean clustering coefficient**	0.78	1.00	0.89	0.87	0.86	0.00	1.00	1.00
**Global efficiency**	0.84	1.00	0.64	0.62	0.69	0.64	1.00	1.00
**Local efficiency**	0.89	1.00	0.95	0.75	0.55	0.00	1.00	1.00

Properties of the full network, plus each principal network containing more than one vertex, are given. The most connected vertex in each case is based on the sum of absolute weights of edges connected to the vertices, ignoring self-connections. Efficiency measures are calculated following Latora & Marchiori [41]. Unweighted versions of all measured are used, where the option exists.

Our bootstrap analysis showed that on average, across the 1000 replicates, 84% of vertex memberships matched for the first principal network, 66% for the second, and 63% for the third. Further, the consensus first PN matched the first PN obtained from the full dataset almost perfectly, with only a single extra vertex appearing in the former.

**Figure 3:**
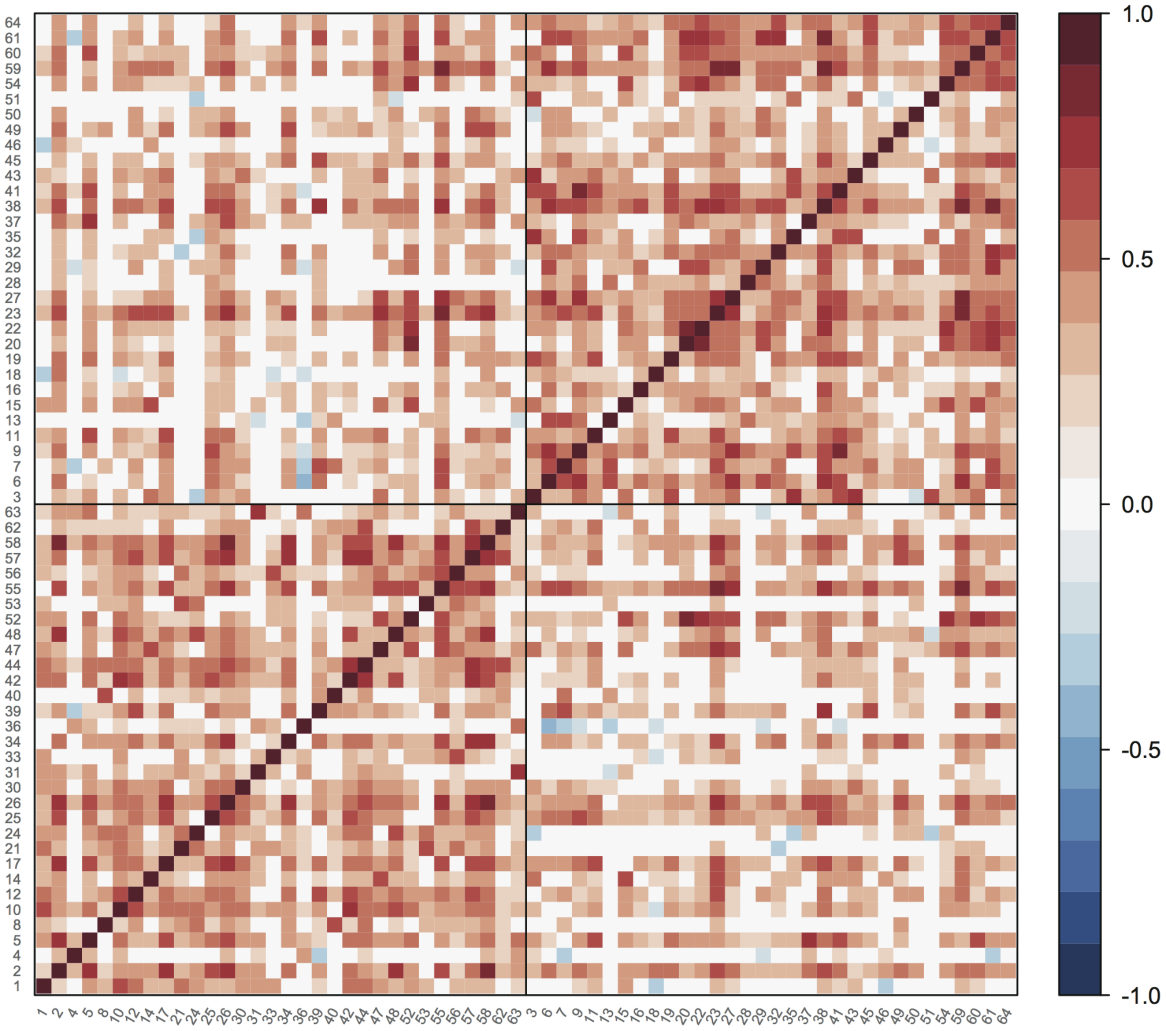
Result of partitioning the thresholded cortical thickness association matrix, using a well-established modularity maximisation algorithm. Two groups of vertices, of equal size, emerge. These are delimited by horizontal and vertical black lines. Since this algorithm does not identify an ordering for vertices, they are ordered numerically within each group.

Running the Newman modularity maximisation algorithm on our cortical thickness data resulted in a partition of the graph into two groups (or “communities”) of 32 vertices, as shown in Fig. 3. Approximately half of the vertices in each group are from each hemisphere. 12 bilateral pairs appear within each of the groups, while the remaining eight, including the precuneus, straddle the partition, with the left-sided region appearing in one group and the right-sided region appearing in the other. There is very little correspondence with the main principal network: 25 of the vertices that appeared in PN1 are in one of the communities, while the other 19 are in the other.

**Figure 4:**
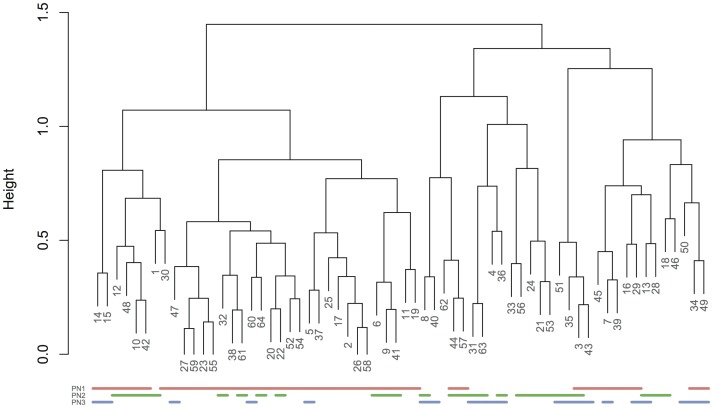
Dendrogram showing the results of hierarchical clustering applied to the cortical thickness data, using (1-correlation) as the distance measure. “Height”, on the *y*-axis, refers to the maximum distance between vertices in each pair of clusters. The coloured lines at the bottom of the figure indicate which vertices appear in each of the three main PNs.

Fig. 4 shows the results of applying agglomerative hierarchical clustering to the cortical thickness data. The pair of vertices with the smallest distance (i.e. highest correlation) is the left and right superior parietal gyrus, followed by the left and right superior frontal gyrus, and then the left and right precuneus. To highlight the relationship between these results and the output of the principal networks analysis, the nodes which appear in each PN are highlighted underneath the dendrogram. We observe that in this case there is an approximate correspondence between one of the top-level clusters and the first PN, although the association is not exact. There is no clear link between clusters and the other two PNs.

**Figure 5:**
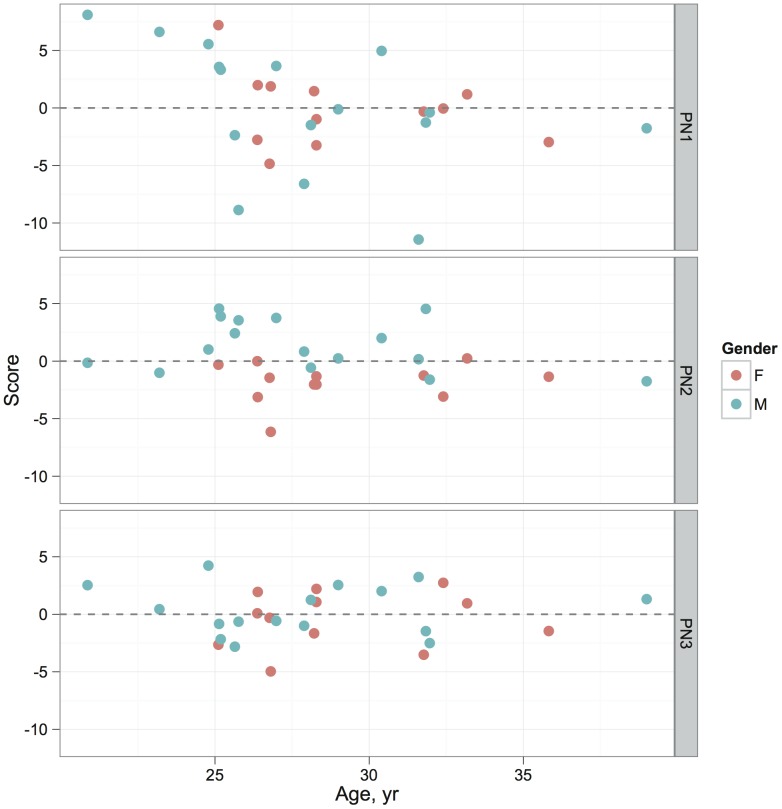
Scores for each of the three major principal networks based on cortical thickness, plotted against age. Note that the scores for each PN sum to zero.

Scores for the first principal network, calculated according to Eq. (4), were very strongly correlated with the mean cortical thickness across all regions (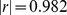, 95% confidence interval 0.962–0.992). No such relationship existed for other major PN scores. Fig. 5 shows plots of the scores for each of the first three PNs against age, by way of illustration of how these values may be used in further analysis. Linear model fits to these data showed that PN1 scores were significantly affected by age but not gender (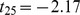, 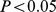); PN2 scores were significantly influenced by gender but not age (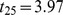, 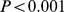 for males relative to females); and PN3 scores were not affected by either variable (both 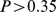). We can interpret the significant effects as differences in the average cortical thickness within the regions appearing in each subnetwork, between the sexes or with age.

**Figure 6:**
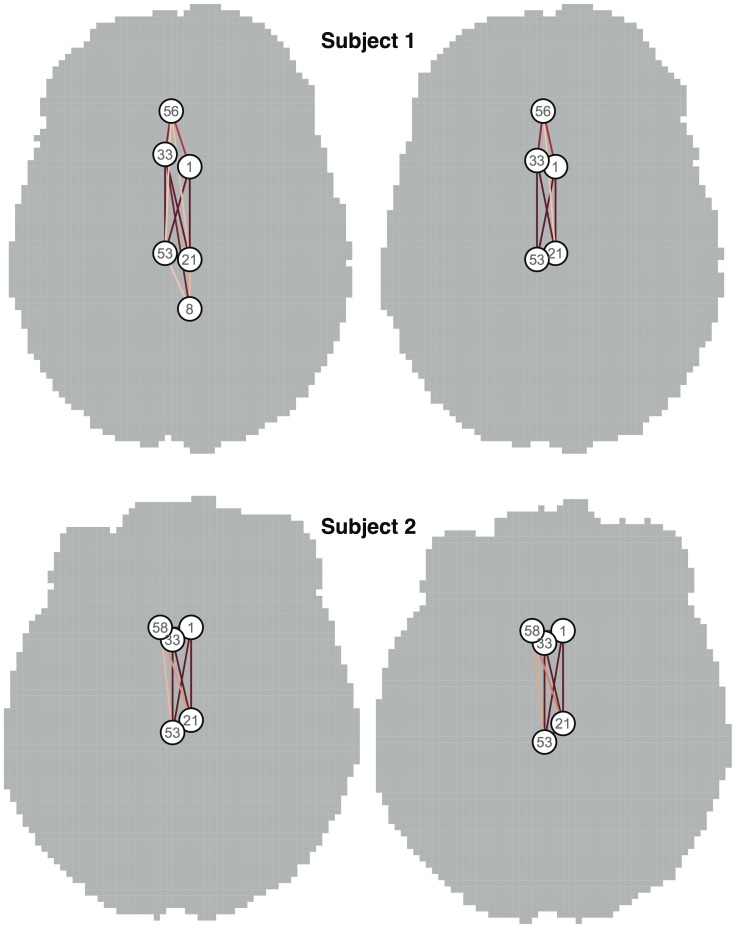
First principal network derived from diffusion data in each of two repeat scans of two subjects. The location of each vertex is based on the original segmentation, using the spatial median of the corresponding region. All four graphs emphasise the strong interconnections between subregions of cingulate cortex.

To illustrate the results obtained subject-by-subject from the diffusion tractography data, the PNs with the largest eigenvalue for each of the two repeated scans of the two individuals with median age (both male) are shown in Fig. 6. In all cases these networks highlight the strong structural interconnectivity between subregions of cingulate cortex: the left and right caudal anterior cingulate cortex, and left and right posterior cingulate gyrus, invariably appear in all four graphs. Graph properties of PN1 in each of these cases are given in Table 3. Region 33, the right caudal anterior cingulate cortex, is the most strongly connected vertex in all cases.

**Table 3:** Graph characteristics of diffusion tractography networks.

Subject	1		2	
**Scan**	1	2	1	2
**Eigenvalue**	10.50	11.01	11.93	12.10
**Number of connected vertices**	6	6	6	5
**Most connected vertex**	33	33	33	33
**Number of edges**	16	18	18	14
**Connection density, %**	76.19	85.71	85.71	93.33
**Mean absolute edge weight**	1.47	1.53	1.65	1.99
**Mean shortest path, steps**	1.20	1.07	1.07	1.00
**Mean clustering coefficient**	0.85	0.93	0.93	1.00
**Global efficiency**	0.90	0.97	0.97	1.00
**Local efficiency**	0.93	0.97	0.97	1.00

Properties of the first principal network are given for each of the diffusion data sets analysed. The most connected vertex in each case is based on the sum of absolute weights of edges connected to the vertices, ignoring self-connections. Efficiency measures are calculated following Latora & Marchiori [41]. Unweighted versions of all measured are used, where the option exists.

**Figure 7:**
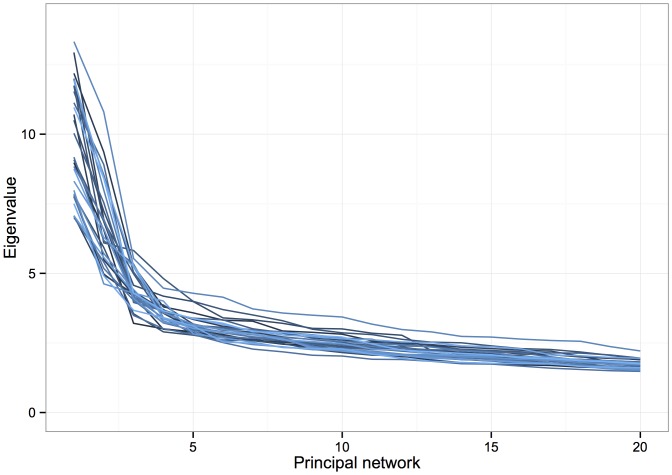
Eigenvalues of the first 20 principal networks derived from each subject's first diffusion MRI data set. The pattern of fall-off is very similar from subject to subject, indicating consistency in subnetwork weights across our cohort.

Across the whole data set, the principal networks were extremely consistent between the first and second scans, with some 97% of vertex memberships agreeing across scans for the first principal network, 96% for the second, and 93% for the third. Moreover, regions of the cingulate cortex were involved in 100% of first PNs across both scans, thereby lending very substantial weight to the importance of these regions in the connectome. The 20 largest eigenvalues for each subject's first scan are shown in order in Fig. 7, and a very similar pattern of fall-off may be observed from subject to subject.

## Discussion

In this manuscript we have described a simple and general technique based on what we refer to as “principal networks”. This method allows brain connectivity networks to be decomposed into subnetworks of strongly interconnected vertices. The influence of each subnetwork in the source data, and the weight of each vertex within a subnetwork, can be obtained easily. In the case of networks based on region-to-region correlation across subjects, such as cortical thickness correlations, the method is functionally equivalent to principal components analysis, and scores for each PN can be calculated subjectwise and compared. We have demonstrated that PNs are stable, meaningful and reproducible: stable, because key aspects of the full network are preserved in major PNs (Figs 1 and 2, Table 2); meaningful, because regions involved in major PNs are consistent with current understanding of the most connected parts of the brain (Tables 2 and 3); and reproducible, because the most important subnetwork obtained is highly consistent across scans (Fig. 6 and Table 3). We have also shown that principal networks can be derived from imaging data of different sorts. Although the spectral decomposition of a graph's association matrix has been well-studied in theoretical work—indeed, it is central to the subfield of spectral graph analysis [21] —we believe that this study represents the first use of our particular approach to identify subnetworks in the context of brain connectivity analysis. Spectral graph methods have however been used in other contexts, such as in disease progression modelling [22], or in the “normalised cuts” algorithm used in image segmentation [23].

In Fig. 1 we demonstrated that the loadings obtained from the principal networks analysis (PNA) reflect real structure in the original association matrix. Fig. 2 showed examples of how the different principal networks capture distinct aspects of the source data, with varying numbers of connected vertices. The first PN by definition represents the subnetwork with the greatest extent of internal connectivity, and as such it was seen to capture the tendency for large-scale all-to-all association in the cortical thickness data, with scores that capture essentially the same information as the overall mean cortical thickness—and correlate with age (see Fig. 5). Subsequent PNs captured more subtle characteristics, with the second PN's scores correlating with gender in this data set. Figs 3 and 4 showed the results of modularity maximisation and hierarchical clustering on the same data for comparison. In Fig. 6, the applicability of the technique to diffusion data was demonstrated, as well as the scan–rescan reproducibility. As Tables 2 and 3 highlight, graph-theoretical measures can be easily derived from all PNs.

In exploratory analyses, where the set of key regions of interest may not be known in advance, and in other cases where multiple circuits may be of relevance, subnetwork analysis of the sort offered by PNA is of significant importance. The key difference between PNA and more well-established community finding approaches such as modularity maximisation is that the former does not simply attempt to partition the graph into subcomponents, but rather factorises the association matrix and constructs separate graphs from each component. This approach has the key advantages of taking all connection strengths into account and allowing different “layers” of association to be extracted. The modularity maximisation approach, by contrast, is a purely graph-based method, and does not consider how strongly vertices are associated. Modularity methods usually do not allow a vertex to belong to multiple communities either, although some recent developments offer more flexibility in this regard [24], [25]. Hierarchical clustering offers another alternative approach to identifying connected subnetworks, and because it considers connection strength, it produces results more closely related to those of PNA (see Fig. 4). Nevertheless, this approach has its own limitations. Clusters are built up from pairwise associations, without reference to the rest of the association matrix. The approach requires a vertex to be either in or out of a cluster, rather than allowing for weights or degrees of uncertainty. For a given “height” threshold, every vertex will appear in exactly one subnetwork: the possibility that a vertex should be wholly disregarded, or that it is involved in several networks, does not exist. Finally, a decision has to be made on how to perform cluster agglomeration (or division), and there are several methods available, including complete linkage, used above, and single linkage, which considers the pair of vertices with the lowest distance. More closely related to the principal networks approach in terms of methodology are spectral clustering and methods based on the eigensystem of the so-called Laplacian matrix of a graph (e.g. [26], [27]), but such methods are usually used to obtain a hard and mutually exclusive partition, tend to be applied to relatively sparse graphs, and are not currently well-used in connectomics. In the end, the best choice of approach is likely to depend on the data and the scientific question under evaluation. PNA simply offers a powerful new alternative, which additionally offers the ability to calculate scores relating to each subnetwork.

The exact results of the principal networks approach will of course depend on the particular measure of interregional association used to create the original graph, and to some extent the nature of other preprocessing steps that are applied to the data. For diffusion data, in particular, a wide range of connectivity measures have been used in the literature, which may attempt to compensate for the sizes of the two regions, their distance from one another, and so on. The association measures we have used in this study are intended to create indicative results to illustrate the method of principal networks, but the method is by no means limited to these, and the choice of measure can be made according to the focus of a particular study. Association matrices which are not positive-semidefinite will produce negative or perhaps even complex eigenvalues, or else not be diagonalisable, but for the vast majority of practical cases with appropriately-chosen association measures, we would expect the method to be applicable. Where noise plays a major role it may be possible to find a positive-semidefinite matrix which closely approximates the observed association matrix [28].

When performing PNA a decision must be made on how many principal networks to consider. A common approach in PCA is to retain those components which have an eigenvalue greater than the value which would be expected if the variance were divided equally amongst all components, and that approach can also be taken with principal networks, but in the graph context we have simply considered networks containing at least two vertices—since smaller networks cannot encapsulate any interesting connectivity information. Less straightforward is the decision on which vertices to include in each subnetwork. In this study we have simply used a loading threshold, the choice of which is inevitably arbitrary. More sophisticated approaches to identifying “significant” loadings have been proposed, such as using bootstrap methods [29], but these introduce additional complexity, and exploration of such techniques is therefore left as future work. Nevertheless, we have shown that using a single threshold level, reproducible results can be obtained. The thresholds were chosen before the analysis began, and no tweaking or optimisation was necessary.

If the analysis is carried out on separate data sets, the question arises of how to identify equivalent PNs from each set of results. Assuming that the full set of vertices is the same in each case, there are a number of ways that this may be achieved, such as by matching up the component eigenvectors to maximise their inner products, which would be computationally straightforward.

One limitation of the principal networks approach laid out above is the constraint that the loading vectors for each subnetwork be orthogonal. In practice, groups of vertices which do not meet this criterion may be of significance, and future work will therefore explore the use of nonorthogonal loadings.

In conclusion, “principal networks” represents a new approach to graph-based neural connectivity analysis, offering a simple and robust way to identify and analyse important subnetworks. It is applicable to a large class of connectivity graphs relevant to clinical and nonclinical neuroscience applications, and provides a platform for investigating the involvement of various neural circuits in populations of interest. A reference implementation of the method will be made available through the open-source TractoR software package [30].

## Methods

### Ethics statement

Informed written consent was obtained from all subjects participating in this study. Processes for consenting and image acquisition were approved by the UCL Research Ethics Committee.

### Theory

In this section we outline the theoretical basis of principal networks. We begin by assuming that some kind of parcellation process has been applied to a structural image of the brain, resulting in a set of regions to be used as the full set of vertices in the connectivity graph. In addition, we assume that some measure of association has been calculated between each pair of regions, resulting in a square association matrix providing some information on the “connection” between regions. Nonsymmetric matrices correspond to “directed” graphs, while an “undirected” graph will have a symmetric association matrix.

We denote the association matrix by 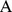. Its elements, 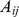, quantify in some sense the connection between regions 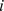 and 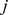, of which there are 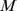 in total. These elements may be binary— i.e. either 1 or 0—if appropriate. Diagonalising the association matrix, we have
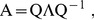
(1)


where 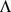 is a diagonal matrix of eigenvalues, 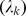, and 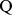 is a matrix whose columns contain the 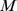 eigenvectors of 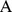. This diagonalisation will be possible in most practical cases, and in particular will always exist for symmetric real association matrices. We note that in the specific case where 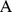 is a correlation matrix, as in cortical thickness or functional connectivity analyses, 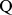 corresponds to the loading matrix of a PCA transformation of the original data—centred and scaled to have zero mean and unit variance within each region—with the 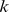
th column of 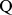 representing the “loadings” for the 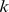
th principal component.

The magnitude of each eigenvalue indicates the degree of influence which the corresponding component had in the original association matrix. However, to form a principal network, we need an association matrix specific to each of these components. We can calculate a “partial” association matrix using Eq. (1), by setting all eigenvalues except the one of interest to zero, viz.
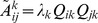
(2)


The full association matrix is then a straightforward sum of these component matrices:
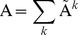
(3)


The loading, or influence, of region 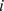 on the 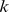
th principal network is given by 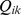, and a threshold may be applied to these values to determine which regions to retain within a given principal network. Likewise, the importance of each edge between the vertices of the 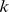
th principal network is given by the appropriate element of the partial association matrix, 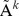.

Finally, in the case where 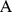 is a correlation matrix, the original 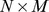 scaled data matrix, 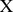
—where 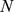 is the number of observations, typically subjects—can be used with the eigenvector matrix to reconstruct a transformed set of “scores”, 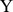, which represent the projection of each data point along the eigenvectors. Hence, we have
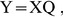
(4)


where 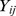 can be thought of as the influence of principal network 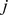 on observation 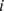. These scores may be meaningfully compared between subsets of the data.

**Figure 8:**
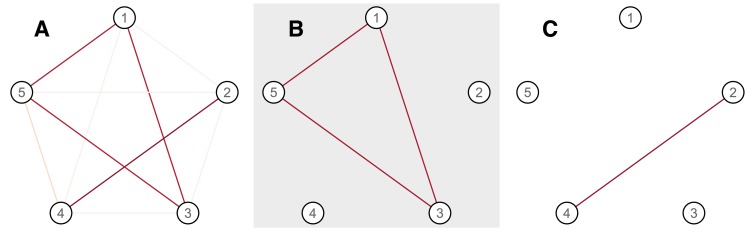
Illustration of the principal networks approach using a simple graph with five vertices (A). The first and second principal networks (B,C) capture the two canonical subnetworks in the graph.

An illustration of the technique applied to a simple network with five vertices is shown in Fig. 8. In this undirected graph, the odd-numbered vertices are connected together with edges of weight 0.8, and the even-numbered vertices are connected together with edges of weight 0.9. All other edges have a weight of 0.05, except for the edge between nodes 4 and 5, which has a weight of 0.2. Self-connections have a weight of unity. It has several characteristics which are typical of brain connectivity networks: its edges are weighted and it is densely connected, although several edge weights are very small.

Principal networks analysis correctly decomposes this graph into the two subnetworks of even and odd vertices. The eigenvalues of the association matrix are, from largest to smallest, 2.65, 1.86, 0.25, 0.20 and 0.05, clearly indicating the presence of exactly two nontrivial eigenvectors. The first eigenvector is strongly weighted on vertices 1, 3 and 5, while the second emphasises vertices 2 and 4. The spectral decomposition of the association matrix captures this information because major eigenvectors represent linear combinations of vertices which jointly make a large contribution to the overall “amount of connectivity” in the full graph.

### Image acquisition and processing

The participants for this study were 28 healthy adults (12 female), with ages ranging from 20 to 39 yr (mean 28.5 yr, standard deviation 3.9 yr). Each participant underwent an MR imaging protocol on a Siemens Avanto 1.5 T clinical system (Siemens Healthcare, Erlangen, Germany), using a self-shielding gradient set with maximum gradient strength of 40 mT m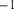, and standard twelve-channel quadrature head coil. Two 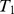-weighted 3D Fast Low-Angle SHot (FLASH) structural images were acquired using 176 contiguous sagittal slices, a 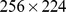 mm field of view and 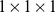 mm image resolution. Flip angle was 15^

^, echo time was 4.94 ms, and repetition time was 11 ms. In addition, echo-planar diffusion-weighted images were acquired for an isotropic set of 60 noncollinear directions, using a weighting factor of 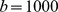 s mm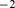, along with three 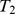-weighted (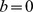) volumes. In this case 60 contiguous axial slices of width 2.5 mm were imaged, using a field of view of 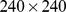 mm and 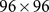 voxel acquisition matrix, for a final image resolution of 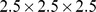 mm. Echo time was 81 ms and repetition time was 7300 ms. The diffusion protocol was performed twice per subject, to allow scan–rescan reproducibility to be assessed.

The 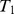-weighted images from each subject were converted from DICOM to MGH format using the TractoR software package [30]. They were then processed using Freesurfer version 5.1.0 to parcellate the cortical surface into 32 gyral regions per hemisphere [31], and to estimate the cortical thickness in each region [32].

Diffusion MRI data from each subject were converted from DICOM to NIfTI-1 format, and preprocessed to remove eddy-current induced distortions. The brain was extracted from the images using the brain extraction tool from the FMRIB Software Library [33]. Diffusion tensors were fitted using standard least-squares estimation, and the fractional anisotropy (FA) calculated at each voxel. A “ball-and-sticks” model, allowing for two fibre orientations per voxel, was fitted for each data set as described by Behrens et al. [34], and whole-brain tractography was performed, initialising one streamline from each voxel with an FA of at least 0.2 to avoid seeding in deep grey matter or cerebrospinal fluid. The exact subvoxel seeding position was subject to random jitter in each case—drawing from a uniform distribution within the voxel—to move the seeds off the regular imaging grid. This allows any part of the voxel to be taken as the seed point, rather than just the centre, thereby avoiding the creation of an unnatural, grid-like streamline set.

### Principal network calculation

Principal networks were calculated from cortical thickness data by calculating the cross-subject correlation matrix between average regional thicknesses, using Pearson's correlation coefficient. This correlation matrix was used directly as the association matrix in this modality, and PNs were calculated as described in the Theory section. A loading threshold of 0.1, ignoring sign, was applied to the vertices of each PN to determine whether or not to include them, and an absolute correlation threshold of 0.2 was used to threshold edges. Scores were calculated for each PN in each subject, following Eq. (4).

For comparison, the well-known and efficient modularity maximisation algorithm described by Newman [35] was applied to the cortical thickness data, after applying the same absolute edge weight threshold of 0.2. This algorithm partitions the graph until further subdivision would not further increase the modularity. In addition, agglomerative hierarchical clustering was performed using 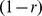 as the distance measure, with 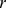 the correlation coefficient between a pair of vertices. A “complete linkage” approach was taken, whereby the distance between clusters is taken to be the maximal distance between pairs of constituent vertices.

The average 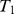-weighted image for each subject, as calculated by Freesurfer, was transformed into the space of the diffusion 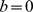 image using nonlinear fast freeform deformation–based registration, as implemented in the the Nifty Reg package [36], using the RNiftyReg interface for R, version 1.0.1 (http://cran.r-project.org/web/packages/RNiftyReg/). Individual cortical regions were extracted from the Freesurfer parcellation and transformed into diffusion space using the same transformation. The number of streamlines passing through each pair of regions of interest was calculated, divided by the average number of voxels in the two regions, and entered into the association matrix as a symmetric metric of connectivity. (A unit value of this metric indicates an average of one streamline per voxel passing through the two regions of interest, a density equal to that used for seeding. Values higher than this are of course possible.) Self-connection values were included so that the association matrix would be positive-definite. PNs were then calculated from this association matrix. A loading threshold of 0.1 was again applied to the vertices of each PN, and a threshold of 0.2 was applied to the connectivity metric.

All analyses were performed and graphics produced using the R software environment for statistical computing [37], [38], along with the “lattice” and “ggplot2” add-on packages [39], [40].

### Bootstrap analysis

In order to estimate the stability of the principal network decomposition of our cortical thickness graph, we performed a bootstrap resampling analysis. For each of 1000 replicates, 28 sets of cortical thickness measurements were obtained by randomly sampling from the 28 subjects with replacement, and the decomposition was repeated. The proportion of regions whose membership in each principal network agreed with that obtained from the original data set was then calculated. A consensus first PN was also calculated by identifying those regions which appeared in the first PN for a majority of bootstrap replicates.
